# Brain–Bone Axis in Physiological and Pathological Conditions

**DOI:** 10.3390/ijms26199822

**Published:** 2025-10-09

**Authors:** Luca Massaccesi, Massimiliano Marco Corsi Romanelli, Emanuela Galliera

**Affiliations:** 1 Department of Biomedical Sciences for Health, Università degli Studi di Milano, 20133 Milan, Italy; mmcorsi@unimi.it (M.M.C.R.); emanuela.galliera@unimi.it (E.G.); 2 IRCCS Ospedale Galeazzi-Sant’Ambrogio, 20157 Milan, Italy; 3 Department of Experimental and Clinical Pathology, IRCCS Istituto Auxologico Italiano, 20145 Milano, Italy

**Keywords:** brain–bone axis, regulatory mechanisms, neurotransmitters, hormones, Alzheimer’s disease (AD), Parkinson’s disease (PD)

## Abstract

The brain–bone axis has garnered increasing attention over the years, leading to numerous studies that have unraveled the intricate bidirectional communication between the central nervous system (CNS) and skeletal metabolism. This review explores this profound relationship, examining the complex mechanisms that regulate it, the key players involved, and the clinical implications of its dysfunction in various pathological situations affecting the CNS and skeletal system. Ultimately, it emphasizes the potential of ongoing research to develop diagnostic tools, therapeutic interventions, and preventive strategies aimed at enhancing skeletal and neurological health.

## 1. Introduction

Recent studies have challenged the traditional view of the skeleton and the central nervous system (CNS) as distinct entities with separate functions within the human body. These studies have introduced the concept of the brain–bone axis [1,2], a bidirectional communication network between the brain and the bones. This network renders the conventional view of the skeleton as a mere structural support system and calcium reservoir incomplete [3,4].

The brain–bone axis encompasses a diverse range of signaling pathways, neuroendocrine factors, and molecular mediators that play a crucial role in regulating bone remodeling through various neural pathways. Recent evidence has shed light on the remarkable ability of bone to function as an endocrine organ, establishing intricate interactions with the brain and other organs in the body [5]. Notably, certain bone-derived factors, such as osteocalcin (OC) [6,7] and fibroblast growth factor 23 (FGF-23) [8], have demonstrated the potential to influence brain function and behavior. These factors can cross the blood–brain barrier (BBB), thereby modulating cognitive processes, including memory and mood.

The brain–bone axis has far-reaching clinical implications, affecting a wide range of skeletal and neurological disorders. Dysregulation of its signaling pathways may contribute to the pathogenesis of conditions like fractures, osteoporosis, and affective disorders such as bipolar disorder (BD) and major depressive disorders (MDD) [9,10,11]. Moreover, studies in both patients and animal models have established a link between bone disease and neurodegenerative diseases like Alzheimer’s disease (AD) or Parkinson’s disease (PD), suggesting a reciprocal interaction between bone and brain in pathology [12,13,14].

Despite these promising findings, research in this area is still in its infancy, and much remains to be understood about the specific mechanisms involved. The implications for therapeutic strategies are enormous. For instance, targeting the brain’s influence on bone health could open new avenues for preventing osteoporosis or treating fractures in individuals with neurological diseases.

The brain–bone axis emerges as a promising frontier in biomedical research, highlighting the interconnectedness of our body systems. As our knowledge advances, it has the potential to lead to innovative therapies that enhance both mental and bone health, ultimately improving the quality of life for many individuals.

This review aims to explore the intricate relationship between the CNS and skeletal metabolism, examining the complex mechanisms that regulate it. It analyzes the key players involved in this relationship and discusses the clinical implications of its malfunctions in various pathological situations affecting the skeletal system and CNS.

## 2. Brain–Bone Axis and Bone Metabolism Key Players

The brain–bone axis, which comprises various signaling pathways, neuroendocrine factors, and molecular mediators, facilitates communication between the CNS and bone metabolism. The CNS exerts significant control over bone remodeling through several neural pathways, including the sympathetic nervous system, hypothalamic neuropeptides, and neurotransmitters [15]. This regulation also operates in the opposite direction. Indeed, bone-derived factors such as OC and FGF-23 have been shown to influence brain function and behavior by crossing the BBB and modulating cognitive processes, memory, and emotional responses. Bone morphogenetic proteins (BMPs) play a crucial role in regulating the growth and differentiation of neuronal cells [16]. Moreover, bone marrow mesenchymal stem cells (BMSCs) have demonstrated the ability to repair damaged neural tissue [17], underscoring the significance of the brain–bone axis in neural regeneration and repair.

**Figure 1:**
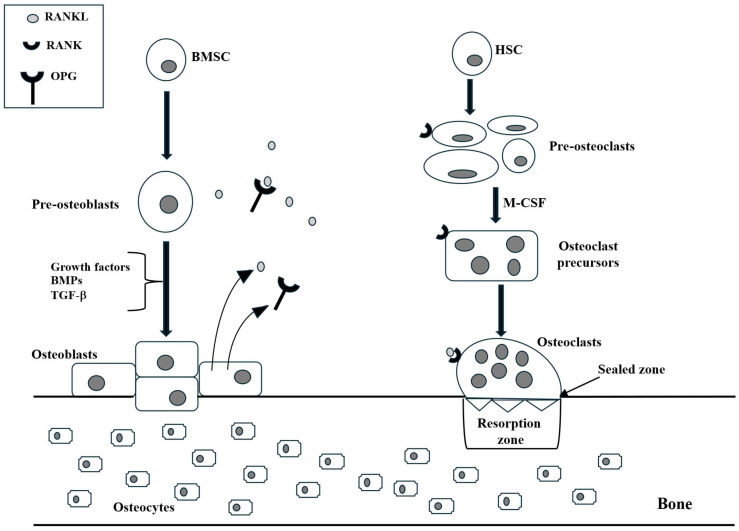
Osteoblast and osteoclast differentiation; RRO axis in bone remodeling regulation. Osteoblasts, originating from bone marrow stem cells (BMSCs), differentiate into osteoblasts under the influence of growth factors such as bone morphogenetic proteins (BMPs) and transforming growth factor b (TGF-b). These cells synthesize and secrete bone matrix components, gradually maturing the bone matrix through mineralization to form new bone tissue. In contrast, osteoclasts, originating from hematopoietic stem cells, differentiate into osteoclasts through the action of factors such as macrophage colony-stimulating factor (M-CSF) and receptor activator of nuclear factor-kB ligand (RANKL). Osteoclasts dissolve the bone matrix by secreting acidic substances and proteases, releasing minerals such as calcium and phosphorus, and facilitating bone tissue resorption. RANKL, secreted by osteoblasts, stimulates RANK-bearing osteoclast precursors to differentiate into active bone-resorbing osteoclasts. This negative feedback loop is essential for physiological bone remodeling, maintaining the delicate balance between bone formation and resorption. OPG, secreted by osteoblasts, acts as a decoy receptor for RANKL, preventing its interaction with RANK and thereby blocking osteoclast maturation. This protective mechanism safeguards bones from excessive osteoclast-mediated resorption.

Bone metabolism encompasses two primary processes: bone formation and bone resorption. These processes are continuous and involve the formation and renewal of bones throughout the human body [18]. Bone formation is a process carried out by osteoblasts, which are derived from BMSCs and differentiate into osteoblasts when stimulated by growth factors like BMPs and transforming growth factor-b (TGF-b). Osteoblasts synthesize and secrete bone matrix components, such as type-1 collagen, gradually maturing the bone matrix through mineralization to form new bone tissue. In contrast, bone resorption is primarily mediated by osteoclasts, which are derived from hematopoietic stem cells and differentiate into osteoclasts when stimulated by factors like macrophage colony-stimulating factor (M-CSF) and receptor of activated NF-kB ligand (RANKL) (Figure 1). Osteoclasts dissolve the bone matrix by secreting acidic substances and proteases, releasing minerals like calcium and phosphorus, and facilitating bone tissue resorption [19]. These two processes are in a state of dynamic equilibrium, maintaining normal bone structure and function.

## 3. Brain and Bone: Similarities in Physiological and Pathological Conditions

### 3.1. Osteoblasts

As mentioned earlier, osteoblasts are the cells responsible for bone tissue production. These mononuclear cells, with a cuboidal morphology (approximately 15–30 mm in diameter), are responsible for the lamellar appearance of bone. They possess functional morphological features for protein synthesis, including an abundant rugose endoplasmic reticulum, a prominent Golgi apparatus, mitochondria, and various secretory vesicles. Osteoblasts, along with myelinating cells such as oligodendrocytes and Schwann cells, are cells with distinct phenotypes that support tissue-specific functions. The Wnt signaling pathway plays a crucial role in the differentiation of these cell types from the common progenitor mesenchymal stem cell (MSC) [20].

Wnt protein ligands are proteins that, when bound to a specific receptor, initiate multiple intracellular signaling pathways. These pathways actively regulate tissue growth and renewal, as well as the differentiation, proliferation, and proper functioning of the cells they interact with. At the neuronal level, the Wnt pathway plays a crucial role in regulating the behavior of neural stem cells during embryonic development. Subsequently, it influences their proliferation, migration, maturation, and integration into neuronal networks [21].

At the bone level, the Wnt/b-catenin pathway promotes osteoblast differentiation, proliferation, and mineralization. This same pathway also induces the expression of osteoprotegerin (OPG), an osteoclast inhibitory factor in osteoblast-lineage cells to prevent bone resorption [20].

**Figure 2:**
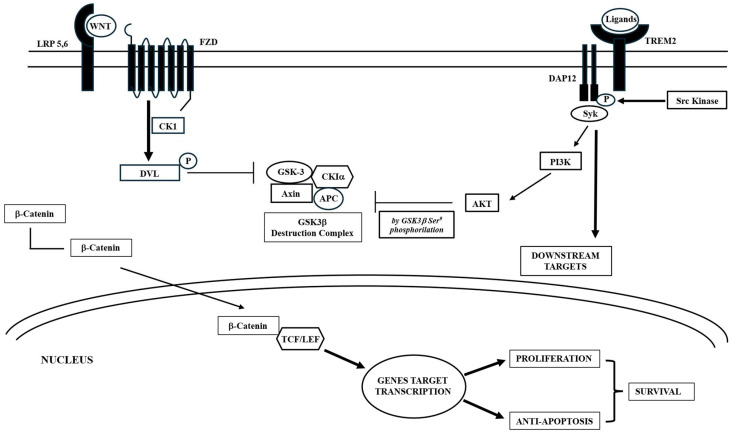
Wnt and TREM2 signaling pathways. Wnt activates the Wnt signaling pathway by binding to frizzled (FZD) and low-density lipoprotein receptor-related proteins 5 and 6 (LRP5/6). This interaction leads to the phosphorylation of disheveled (DVL) proteins by casein kinase-I. Phosphorylated DVL then triggers the degradation of the GSK3β-complex, which normally breaks down β-catenin, causing its dysfunction. As a result, Wnt increases the stability and accumulation of β-catenin in the cytoplasm, facilitating its entry into the nucleus. Once in the nucleus, β-catenin binds to T cell factor/lymphoid enhancing factor (TCF/LEF), promoting the transcription of target genes. Ligand binding to TREM2 induces the phosphorylation of two tyrosine residues in the ITAM motif of DAP12 by Src kinase. Then, Syk kinase is recruited to the ITAM motif to activate downstream targets. TREM2 regulation of b-catenin signaling: Syk kinase activates PI3K/Akt signaling, resulting in the phosphorylation of the serine 9 residue of GSK3, leading to GSK3β-complex inactivation and stabilization of b-catenin.  b-catenin accumulates in the cytoplasm and then enters the nucleus, where it regulates the expression of target genes.

In more detail, at neuronal and bone levels, Wnt activates the Wnt signaling pathway by binding to frizzled (FZD) and low-density lipoprotein receptor-related proteins 5 and 6 (LRP5/6). This interaction leads to the phosphorylation of disheveled (DVL) proteins by casein kinase-I. Phosphorylated DVL then triggers the degradation of the adenomatous polyposis coli (APC)-axin-glycogen synthase kinase 3 beta (GSK3b) complex, which normally breaks down β-catenin, causing its dysfunction. As a result, Wnt increases the stability and accumulation of β-catenin in the cytoplasm, facilitating its entry into the nucleus. Once in the nucleus, β-catenin binds to T cell factor/lymphoid enhancing factor (TCF/LEF), promoting the transcription of target genes (Figure 2).

When the bone lacks this signal, there is a significant increase in both the number and activity of osteoclasts, leading to substantial bone loss. Consequently, alterations in the Wnt signaling cascade are often associated with other events, such as bone loss and the onset of neuropathologies [22,23].

The Wnt/b-catenin pathway plays a crucial role in the onset of AD. Its normal activation helps inhibit the production of amyloid beta (Ab) and hyperphosphorylation of tau (P-tau) in the brain. However, as the brain ages, this pathway tends to become less active, with a more pronounced reduction in the brains of AD subjects [24]. Reduced or absent functionality of the Wnt co-receptor LRP6 has been linked to downregulation of the Wnt/b-catenin signaling pathway, which is associated with an increased risk of developing AD [25]. This lack of functionality contributes to the synaptic dysfunction and amyloid plaque accumulation observed in AD.

Numerous studies conducted in animal models, in vitro, and on samples from human AD patients have demonstrated impaired or absent function of the Wnt/b-catenin signaling pathway. Furthermore, the Wnt/b-catenin signaling pathway is implicated in the development of osteoporosis, a common condition observed in AD patients [22,26].

### 3.2. Osteoclasts

Osteoclasts, the primary cells responsible for bone resorption, are irregularly shaped multinucleated giant cells that arise from the fusion of mononucleated cells derived from hematopoietic stem cells [27] (Figure 1). The fusion of these cells is influenced by various factors, including M-CSF, which stimulates osteoclast proliferation and inhibits their apoptosis, and RANKL, which is expressed by both osteoblasts and osteocytes.

The RANK/RANKL/OPG signaling pathway, also known as the RRO axis, has been a subject of research for the past two decades. This three-part pathway plays a crucial role in both the immune and bone systems. Disruption of this pathway has been linked to various health conditions, including bone and immune disorders, certain cancers, and diabetes [28,29]. Recent research has shed light on the impact of the RANKL/RANK system on specific regions of the nervous system, suggesting its potential relevance in neurological disorders [30]. The RRO system was initially identified through studies demonstrating its role in immunity by acting on dendritic cells (DCs) and bone homeostasis by regulating osteoclasts [31,32]. The RANK system, comprising three main molecules—RANKL, receptor of activated NF-kB (RANK), and OPG—plays a crucial role in osteoclastogenesis. RANKL, secreted by T cells, enhances the immune response by promoting dendritic cell survival and function through RANK signaling [33]. In contrast, RANKL can induce immune tolerance by stimulating the differentiation of regulatory T cells (Treg) in certain autoimmune diseases, such as diabetes mellitus [34]. From these results, it becomes evident that the RRO system can activate or suppress the immune response depending on the specific situation.

In addition to the immune system, the RRO axis plays a crucial role in bone remodeling. RANKL, a glycoprotein secreted by osteoblasts, osteocytes, hypertrophic chondrocytes, and bone marrow stromal cells, stimulates RANK-bearing osteoclast precursors to differentiate into active bone-resorbing osteoclasts. This negative feedback loop is essential for physiological bone remodeling, maintaining the delicate balance between bone formation and resorption. OPG, a glycoprotein composed of 380 amino acids and seven structural domains, is secreted by osteoblasts, bone marrow stromal cells, B cells, and DCs. It acts as a decoy receptor for RANKL, preventing its interaction with RANK and thereby blocking osteoclast maturation (Figure 1). This protective mechanism safeguards bones from excessive osteoclast-mediated resorption. Conversely, an imbalance in RANKL/RANK signaling can lead to various bone diseases, including rheumatoid arthritis and osteoporosis [35,36].

The RANKL/RANK axis plays a crucial role in various mechanisms beyond bone homeostasis. It is essential for immune regulation and numerous related processes, including lymphocyte development, lymph node organogenesis, T-cell and dendritic cell interactions, and thymus development [37]. The RRO system has been demonstrated to promote breast cancer cell migration to the bone and subsequent metastasis. It has been described as accelerating the migration and metastasis of RANK-expressing cancer cells [38].

Recent research has revealed that specific neural tissue cells, including microglia, other resident macrophages, neurons, and oligodendrocyte precursor cells, express components of the RRO system [39]. Activation of microglia is a common feature of neurodegenerative diseases, and understanding how the RRO axis influences this activation offers new avenues for comprehending the underlying mechanisms and developing targeted therapies.

In neurodegenerative disorders like AD, characterized by the accumulation of Ab plaques and neuroinflammation, the RRO signaling pathway may play a pivotal role. The activation of microglia by Ab plaques leads to the release of pro-inflammatory cytokines. OPG, acting as a decoy receptor, can modulate this microglial response by preventing RANKL from binding to RANK, thereby attenuating the inflammatory cascade [40].

OPG, a bone-derived protein, plays a crucial role in microglial activation, which is a key factor in the pathogenesis of AD. PD, another neurodegenerative disease characterized by the loss of dopaminergic neurons and chronic neuroinflammation, also has potential links to RRO signaling. Activation of microglia contributes to the inflammatory milieu in PD, leading to progressive neuronal degeneration. OPG’s influence on microglial responses through RANKL/RANK interactions suggests that it may modulate the neuroinflammatory environment, offering a new avenue for therapeutic intervention targeting both inflammatory and neurodegenerative aspects of PD [41].

In this context, it is noteworthy that Denosumab (dmab) warrants mention. Dmab, a human monoclonal antibody, stands as the most potent antiresorptive agent currently employed in clinical practice. Its primary function is to neutralize RANKL, thereby preventing the binding of the cytokine to its receptor (RANK) and effectively inhibiting osteoclast-mediated bone resorption. FDA-approved for osteoporotic postmenopausal women at high risk of fracture, dmab has demonstrated a significant reduction in the risk of vertebral and nonvertebral fractures compared to placebo. Furthermore, it has been shown to enhance bone mineral density (BMD) [42,43].

However, the most noteworthy development in recent years has been the increasing number of studies that have demonstrated the efficacy of dmab in neuromuscular diseases. A case report of a 14-year-old patient with spinal muscular atrophy II (SMA II) demonstrated a remarkable 19% improvement in lumbar spine bone mineral density (LS BMD) after 6 months of receiving 60 mg subcutaneous doses of dmab without any adverse effects [44]. Similarly, a 13-year-old boy with Duchenne muscular dystrophy (DMD) and gastrointestinal obstruction received the same dosage of dmab, which resulted in a significant 16% increase in LS BMD at 12 months [45]. Furthermore, dmab has been shown to prevent the worsening of cardiac hypertrophy in mice and appears to have reduced cardiac hypertrophy in two children with DMD [46].

As seen above, osteoclasts and microglia cells originate from hematopoietic stem cells. They play a crucial role in regulating their microenvironments by eliminating waste materials and ensuring the proper functioning of the relevant tissues. Both cell types utilize common growth factors and signaling molecules. Among the various regulatory pathways shared by these two cell types, the Triggering Receptor Expressed on Myeloid Cells 2 (TREM2) pathway stands out. TREM2 is a transmembrane glycoprotein of approximately 40 kDa (reduced to 26 kDa after N-deglycosylation) that belongs to the single immunoglobulin variable (IgV) domain receptor family [47,48]. The two best characterized members of this receptor family, TREM1 and TREM2 [49,50], exert their functions upon binding to a DAP12 (a member of the type I transmembrane adapter protein family), which mediates their signaling pathway [51].

TREM2 plays a primary role in regulating the functions of three distinct cell types, all of myeloid origin: microglia, osteoclasts, and immature DCs. In the CNS, TREM2-DAP12 forms a signaling complex exclusively expressed in microglia. TREM2, a surface receptor on microglia, activates pathways vital for microglial cell function, including phagocytosis, debris clearance, and survival. Ultimately, TREM2 signaling is essential for maintaining CNS tissue homeostasis [52].

It is important to note that this pathway collaborates with other regulatory pathways. A recent study revealed that TREM2 promotes microglial survival by activating the Wnt/β-catenin signaling pathway (Figure 2) [53], which plays a crucial role in various biological processes [54]. TREM2 activation leads to DAP12 phosphorylation through Src family kinases, initiating downstream signaling cascades [55], which lead to various effects, including the phosphorylation of a specific Ser residue of the GSK3β complex, resulting in its inactivation and consequent stabilization of β-catenin. Conversely, TREM2 deficiency results in a significant downregulation of Wnt/β-catenin signaling accompanied by decreased microglial survival and increased cell death [53]. TREM2 actively opposes the formation of Ab plaques typical of AD. Indeed, it has been shown that this protein specifically binds to Ab, particularly to its soluble oligomers, small aggregates of Ab. This prevents them from aggregating to form polymers that precipitate and give rise to plaques. Moreover, the absence or low level of TREM2 proteins interferes with the proper functioning of the potassium ion channels in microglial cells, reducing their activation and consequently their ability to prevent the formation of Ab plaques or clear them [56].

Nasu–Hakola disease, also known as polycystic lipomembranous osteodysplasia with sclerosing leukoencephalopathy (PLOSL), is a remarkable example of how alterations in the bone-brain axis profoundly impact and intertwine at both the bone and neurological levels. This unique disorder is characterized by the presence of multiple bone cysts, accompanied by a distinctive form of neurodegeneration that leads to dementia and premature death, typically occurring in the fifth decade of life. The cause of Nasu-Hakola disease lies in mutations in the genes encoding DAP12 and TREM2, resulting in the nonfunctioning of either DAP12 or TREM2. Consequently, this disruption of the proper TREM2/DAP12 signaling pathway affects both the CNS and osteoclasts, leading to a cascade of neurological and clinical bone manifestations [57,58]. At the brain level, the absence of the TREM2/DAP12 pathway results in microglial dysfunction, impairing their function and potentially leading to premature microglial apoptosis. This, in turn, triggers a series of brain damage and functional impairment, ultimately causing dementia. On the bone level, the absence of the TREM2/DAP12 pathway leads to osteoclast dysfunction and premature apoptosis. This disruption impairs the bone matrix’s remodeling process, resulting in the formation of bone cysts [59].

Similarly to the research on dmab and RANKL, TREM2 has emerged as a potential therapeutic target. Antibodies targeting the extracellular domain of TREM2 have been identified, capable of stimulating TREM2 signaling through cross-linking [60,61]. These antibodies bind to the extracellular domain of TREM2 and activate downstream signaling pathways, including the phosphorylation of DAP12 and Syk. Preclinical studies have shown that these agonist antibodies improve microglial survival, proliferation, and phagocytosis of myelin debris and Ab.

Research has also focused on the study and development of TREM2 antagonists. Notably, Mirescu and colleagues have highlighted the potential of the antagonist VG-3927 [62]. VG-3927 has been shown to favorably regulate human microglia activation and confer a broad neuroprotective profile in preclinical models. An ongoing clinical trial aims to further investigate the potential of VG-3927 as a therapy for AD via oral treatment.

### 3.3. Osteocytes and Neurons Share Morphological Characteristics

Osteocytes, comprising approximately 90–95% of total bone cells, are the most numerous and long-lived cells in bone tissue. Their lifespan can extend up to 25 years [27]. These cells represent the final stage of osteoblast differentiation. At the end of a bone formation cycle, osteoblasts are incorporated from the bone matrix and settle into the bone gaps [63]. This process involves morphological and structural changes, such as a reduction in the size of osteoblasts, a decrease in the number of organelles like the rough endoplasmic reticulum and Golgi apparatus, and an increase in the nucleus-cytoplasm ratio. Notably, osteocytes possess a characteristic dendritic morphology, closely related to their crucial functions within bone tissue [27].

Osteocytes, irregularly shaped cells with a prominent nucleus and cytoplasm containing numerous extensions, reside in bone lacunae. These lacunae are connected by microscopic canaliculi branching in all directions. Through these canaliculi, the cytoplasmic extensions of different osteocytes communicate with each other via communicating junctions and with blood capillaries present in the bone canals. This allows metabolic exchanges between osteocytes and blood. In essence, these cytoplasmic extensions serve as sensors, detecting changes in the interstitial fluid within bone lacunae and canaliculi. Consequently, osteocytes adapt to these changes and mechanical stresses through various regulatory factors that influence bone dynamics, including deposition and resorption processes.

Among these factors, FGF-23 should undoubtedly be mentioned for its action at both the bone and neuronal levels. It is a growth factor produced by osteocytes and osteoblasts that exerts its effects within the FGF-23/α-KL/FGFR-1 pathway.

α-Klotho (α-KL), a membrane protein encoded by the Kl gene, is primarily expressed in the encephalon (choroid plexuses) and kidney (distal tubules), as well as in the pancreas and other cells such as MSCS, osteoblasts, osteoclasts, and osteocytes. The primary mechanism of action of α-KL is to bind the FGFR-1 receptor, thereby increasing its affinity for the growth factor FGF-23. FGF-23, produced by osteocytes and osteoblasts, is considered a bone-derived hormone that plays a crucial role in bone remodeling processes by regulating mineral homeostasis.

At the renal level, FGF-23 maintains the homeostasis of phosphorus and vitamin D hormone, 1,25(OH)_2_D3, by promoting its reabsorption at the renal tubule. The action of FGF-23 is subject to a feedback regulatory mechanism, which is dependent on phosphatemia levels. This mechanism modulates the expression of klotho. In this regard, it has been [64]. Conversely, in case of increased klotho level, FGF-23 increased significantly, accompanied by hypophosphatemia [65].

FGF-23 activates other pathways at the bone level, mediated by EGR-1, which participates in osteoblast differentiation, and ERK, which plays a crucial role in osteoblast and osteocyte differentiation, as well as mineralization processes. At the neuronal level, FGF-23 promotes neurogenesis and is essential for maintaining cognitive functions [66].

Several studies have demonstrated that elevated serum FGF-23 levels can impair long-term potentiation (LTP) in the hippocampus. This impairment is associated with reduced adenosine triphosphate (ATP) content in the hippocampus, leading to cognitive and memory decline, particularly in individuals with chronic kidney disease [67,68,69].

Studies in animal models, on the other hand, have shown that FGF-23 knockout mice exhibit a severe cognitive impairment, which is independent of the lack of klotho expression. This suggests that FGF-23 plays a crucial role in cognitive function and that its absence can lead to rapid and early onset of cognitive abnormalities reminiscent of premature aging [70]. Therefore, it can be inferred that the action of FGF-23 operates on the edge of a delicate balance, and any disruption in this balance can have severe consequences for cognitive function.

## 4. Regulators of Bone Metabolism—Neurotransmitters and Hormones

The physiological processes of bone metabolism are regulated by various factors, including neurotransmitters, cytokines, hormones, and mechanical stress.

Among the various regulators that connect the brain and bones, circulating factors like leptin, neuropeptide Y (NPY), and semaphorins have gained increasing attention [71]. These molecules can cross the BBB and reach the arcuate nucleus (ARC). Changes in these factors affect the secretion of endocrine factors and the transmission of nerve signals, ultimately impacting bone tissue. It is known that the leptin receptor plays a role in reducing osteogenesis at the bone level [72]. Additionally, leptin is involved in the anti-osteogenic process through central control [73]. These effects are mediated by neural circuits that transmit signals to bone cells through the sympathetic nervous system [74].

The crucial role of NPY in immune responses, endocrine activities, and energy homeostasis is well-established. It is worth noting that the reciprocal effects of NPY and leptin on energy homeostasis suggest the possibility of an interaction in the control of bone formation [75]. This hypothesis was later corroborated by several studies that shed light on the role of NPY as a neural mediator in bone remodeling and homeostasis.

Semaphorins have been shown to be involved in the differentiation and migration of osteoblasts and osteoclasts [76]. Alterations in their expression have been linked to the onset of diseases such as osteoporosis and osteopenia [77].

For instance, parathyroid hormone (PTH) and vitamin D hormone primarily promote bone resorption, while insulin-like growth factor-1 (IGF-1) and estrogen primarily promote bone formation [78,79]. Additionally, bone metabolism is influenced by factors such as genetics, age, sex, diet, and exercise. The equilibrium of bone metabolism is crucial for bone health. If bone resorption surpasses bone formation, it can lead to osteoporosis, causing bones to become brittle and prone to fractures. Conversely, if bone formation exceeds resorption, osteopetrosis can occur, resulting in excessively hard and brittle bones [80]. Therefore, maintaining a normal balance in bone metabolism is essential for preventing and treating bone diseases like osteoporosis.

### 4.1. Leptin

**Figure 3:**
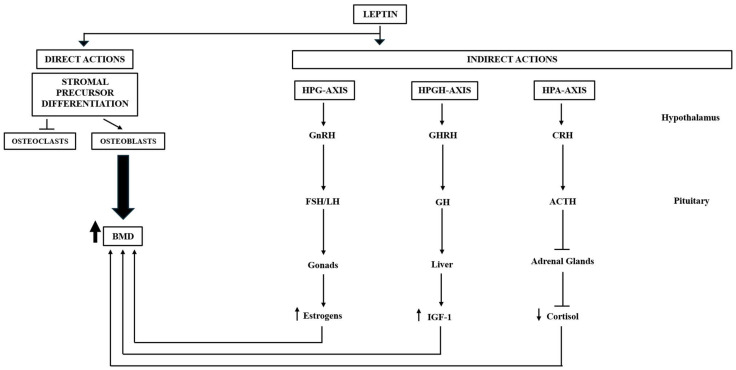
Bone mass regulation by Leptin. Leptin exerts significant influence on multiple hypothalamic-pituitary axes, indirectly impacting target organ systems via their respective hormonal pathways. Consequently, it elicits direct and indirect actions on the bone system. CRH, corticotropin-releasing hormone; IGF, insulin-like growth factor; ACTH, adrenocorticotropin hormone; GH, growth hormone; FSH, follicle stimulating hormone; LH, luteinizing hormone; GHRH, growth hormone releasing hormone; GnRH, gonadotropin-releasing hormone (→ Activation; ⊥ Inhibition; ↑ Levels Increase; ↓ Levels Decrease).

Leptin, a protein hormone with a molecular weight of 16 kDa, produced primarily by adipocytes and encoded by the Ob (Lep) gene, has a critical function in appetite regulation and energy homeostasis. Its actions depend on the level of energy reserves and acute changes in energy balance. Leptin centrally regulates appetite and energy expenditure precisely according to energy availability [81,82]. Over the years, numerous studies have underscored the multifaceted regulatory functions of leptin, particularly its pivotal role in bone metabolism. Leptin exerts its influence on bone mass through both direct and indirect mechanisms (Figure 3)

Directly, it stimulates the proliferation of osteoblasts while simultaneously inhibiting the activity of osteoclasts [83]. In contrast, the indirect action of leptin occurs through the regulation of the hypothalamic–pituitary–adrenal (HPA) and growth hormone/insulin-like growth factor-1 (GH/IGF-1) axes, both of which are essential for maintaining bone homeostasis [84].

Several studies conducted in mouse models (ob/ob mice) have demonstrated how leptin exerts its direct effects on bone at lower doses compared to its metabolic actions. These studies have also revealed that leptin can enhance the perimeter of osteoblasts, mineralize their structures, and facilitate bone formation. Numerous research findings suggest that subcutaneous leptin administration leads to increased mRNA levels of bone and cartilage matrix proteins in the tibiae of mice [85]. Leptin has been shown to regulate osteoblasts by increasing the translation of the Ap-1 gene, which subsequently promotes osteoblast proliferation through the activation of cyclin D1 [86]. Additionally, leptin promotes osteoblast differentiation by upregulating the expression of transforming growth factor-β (TGF-β), IGF-1, collagen-1α, alkaline phosphatase, and OC mRNA in human iliac crest osteoblasts [87]. Other studies have also shown how leptin inhibits osteoclast differentiation by increasing OPG levels. OPG, in turn, binds to RANKL, thereby reducing osteoclast activity [74].

Leptin’s indirect action is exerted through its interaction with various axes that are regulated by the hypothalamic-pituitary-gland network. Leptin interacts with the HPG-axis (hypothalamic–pituitary–gonadal axis) by promoting the release of gonadotropin-releasing hormone (GnRH) [88] at the hypothalamic level, which subsequently leads to increased levels of estrogen that plays a crucial role in bone metabolism.

Leptin, similarly, stimulates the release of GHRH (growth hormone releasing hormone) at the level of the HPGh-axis (hypothalamic–pituitary–growth hormone-axis). GHRH, in turn, stimulates the release of GH, which, at the hepatic level, promotes the production of IGF-1 [89]. IGF-1 is a potent promoter of bone growth and mineralization, acting directly on chondrocytes and osteoblasts. Furthermore, IGF-1 stimulates the synthesis of collagen and other bone matrix proteins, which are essential for the formation of strong and healthy bone.

Leptin appears to have a general inhibitory effect on the HPA-axis (hypothalamic–pituitary–adrenal axis). In vitro studies have demonstrated that cortisol secretion stimulated by adrenocorticotropic hormone (ACTH) decreases in a dose-dependent manner when adrenocortical cells are incubated with leptin [90]. In simpler terms, leptin regulates the secretion of cortisol by the adrenal glands by reducing its production. Consequently, the resulting decrease in cortisol levels promotes bone mass deposition (Figure 3).

Given the intricate and multifaceted role of leptin at the bone and neuronal levels, it becomes evident how alterations in its levels can have profound consequences at both neurological and bone levels. An illustrative condition is obesity, characterized by excessive fat accumulation, significantly impacts metabolic health. Beyond its well-known association with cardiovascular disease, diabetes, and other metabolic disorders, recent studies have uncovered a link between obesity and neurodegeneration [91,92].

The primary cause of this link is the inflammation induced by the growth of adipose tissue, which initiates detrimental processes that adversely affect brain function. Adipose tissue, particularly that surrounding organs, releases various substances that induce inflammation, including cytokines (TNF-α, IL-6), adipokines, and free fatty acids. These substances contribute to persistent, low-grade systemic inflammation, which is increasingly acknowledged as a substantial factor in peripheral metabolic dysfunction and CNS pathology.

In the context of obesity-related inflammation, resident immunocells within the brain, microglia, undergo hyperactivation, producing neurotoxic substances that can induce neuronal death. This neuroinflammation further exacerbates the detrimental effects of obesity on brain health, elevating the risk of cognitive decline, AD and other neurodegenerative disorders.

As previously discussed, leptin plays a pivotal role in regulating appetite and preserving energy homeostasis. Its actions are influenced by the body’s energy reserves and abrupt fluctuations in energy balance. Generally, an increase in adipose tissue leads to a surge in leptin levels, which signals the brain that the body has adequate energy reserves [93]. However, leptin resistance occurs when the brain becomes less receptive to leptin signals despite elevated blood levels [94]. This can manifest due to leptin’s inability to reach the hypothalamus due to a diminished capacity to traverse the BBB, or because there are alterations in the expression and/or sensitivity of leptin receptors [95]. Leptin resistance is exacerbated by persistent low-grade inflammation, a common characteristic of obesity. Pro-inflammatory cytokines such as TNF-α and IL-6 disrupt leptin signaling pathways, particularly in the hypothalamus, impairing appetite regulation and affecting other pathways where leptin exerts regulatory influence. Consequently, alterations in leptin or its receptors may be associated with neurological disorders. Clinical studies have demonstrated an inverse correlation between circulating leptin levels and the risk of AD, strongly suggesting a protective role of leptin in preventing the onset of this disease. Indeed, it is crucial to highlight how the accumulation of hyper-phosphorylated tau and Ab leads to impairments in hippocampal excitatory synaptic function, ultimately resulting in cognitive deficits [96,97]. Numerous studies conducted over the years in animal models have unequivocally demonstrated how leptin exerts beneficial and protective effects on CA1 synapses within the hippocampus [98]. Furthermore, leptin has, more generally, neuroprotective effects at CNS level [99].

In essence, the chronic inflammatory state associated with obesity, which is widely regarded as a potential risk factor for AD [100], also contributes to the phenomenon of leptin resistance. This resistance on the one hand leads to a reduction in bone mass while on the other hand impairs neuroprotection against the detrimental effects of the accumulation of hyper-phosphorylated tau and Ab, ultimately leading to the development of cognitive impairments.

Leptin, a drug already licensed for the treatment of obesity, has demonstrated remarkable efficacy. In the early 2000s was showed that leptin administration can also induce pro-cognitive effects, as evidenced by increased gray matter volume in functional brain imaging. Subsequently, a study reported significant cognitive improvements in a 5-year-old child with congenital leptin deficiency following leptin treatment [101]. This study demonstrated that leptin therapies can effectively cross the BBB, reaching key brain regions involved in cognitive processes, such as the hippocampus.

This evidence served as the foundation for a series of studies conducted in subsequent years, which yielded intriguing results, such as the development of small leptin-based peptides. These peptides hold potential therapeutic value for the treatment of AD. For instance, Malekizadeh and Doherty’s research [102,103] identified small leptin fragments, particularly four short amino acid hexamers, which exhibited the beneficial effects of leptin. In cellular models of AD, treatment with these bioactive hexamers effectively mitigated the acute adverse effects of Ab on hippocampal synapses. Furthermore, they demonstrated the ability to limit neuronal cell death and tau phosphorylation induced by chronic Ab exposure [102]. Based on these findings, leptin-based small molecules emerge as promising candidates for therapeutic use in AD treatment. While these discoveries offer some optimism, substantial experimental evidence is still necessary before leptin-based molecules can be advanced for clinical trials.

Other pathological conditions that significantly involve leptin include anorexia nervosa, a severe mental illness and eating disorder, and hypothalamic amenorrhea. In individuals with anorexia nervosa, leptin levels experience a sharp decline, leading to deterioration of microarchitecture and bone structural integrity [104].

Similarly, women with hypothalamic amenorrhea have also demonstrated a substantial drop in leptin levels. This decline results in a reduction in BMD and an increased risk of bone fractures [105].

In the case of hypothalamic amenorrhea, leptin administration has been shown to be effective. It leads to increased levels of IGF-1, estrogen, and thyroxine, which have beneficial effects on bone mass and integrity. This is evidenced by increased expression of markers of bone formation, such as OC and alkaline phosphatase [106].

### 4.2. Neuropeptide Y

NPY, a 36-amino acid protein, is widely expressed in the mammalian brain. As previously discussed, NPY plays a pivotal role in immune responses, endocrine functions, and energy homeostasis. Numerous studies have underscored NPY’s role as a neural mediator in bone remodeling processes and the maintenance of bone homeostasis [75,107]. Overexpression of hypothalamic NPY has been associated with antiosteogenic effects in the neural tissue of the ARC [108,109]. Conversely, NPY-deficient mice exhibit increased bone volume and osteoblastic activity, as observed by Baldock and colleagues [110].

**Figure 4:**
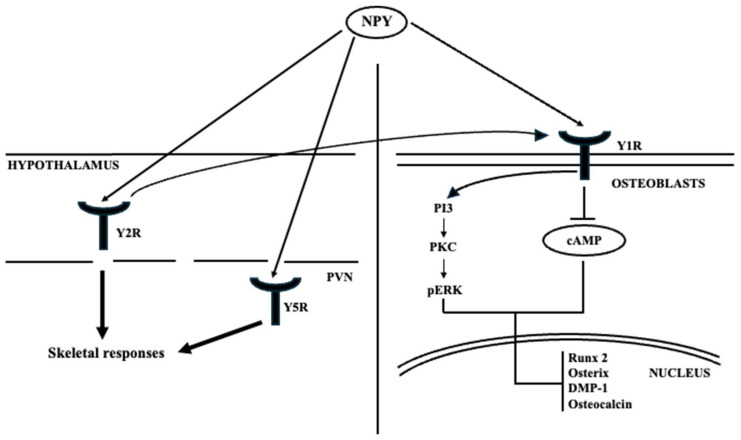
NPY as regulators of bone homeostasis. In the hypothalamus, NPY can regulate skeletal responses through Y2R and Y5R (expressed in the PVN). The Y2R-dependent signaling pathway also has an additional action on bone homeostasis by regulating the expression of Y1R by osteoblasts. In osteoblasts, NPY activates Y1 receptors, inhibiting cAMP signaling and activating PKC with consequent ERK phosphorylation. These events suppress osteoblastic differentiation. cAMP: cyclic adenosine monophosphate; ERK: extracellular signal-regulated kinase; NPY: neuropeptide Y; PI3: phosphatidylinositol-3-kinase; PKC: protein kinase C; PVN: paraventricular nucleus.

NPY exerts its catabolic effects on bone homeostasis by binding to its specific receptors, the Y receptors, which are a family of G-protein-coupled receptors categorized into five subtypes: Y1R, Y2R, Y4R, Y5R, and Y6R. These receptors are specifically localized in various central and peripheral tissues. Thus, NPY regulates bone homeostasis mainly through Y2R and Y5R expressed in the hypothalamus (more specifically, Y5R in PVN) or Y1R expressed in osteoblasts (Figure 4).

Extensive research was conducted on YR-knockout mice, demonstrating that the absence of expression of these receptors in their respective regions (hypothalamus and osteoblasts) resulted in increased bone volume, confirming the catabolic effects of NPY on bone homeostasis [111]. These studies have elucidated the mechanisms by which downregulation or inhibition of Y1R can augment osteogenesis in diverse ways. Firstly, downregulation of Y1R facilitates osteogenesis in bone marrow mesenchymal stem cells (BMSCs) via the cAMP/PKA/CREB pathway, ultimately leading to an enhancement of bone mass. Secondly, it stimulates osteoblastic activity and osteogenic potential [112].

Several studies have investigated the effects of deleting the Y2 receptor (Y2R) on osteoblasts and osteoclasts. Deletion of Y2R increases osteoblastic activity, leading to an increase in the expression of osteogenic transcription factors Runx2 and Osterix. This, in turn, reduces the number of osteoclasts, thereby decreasing bone loss [113]. Baldock et al. compared double (Y1-/- Y2-/-) and single (Y1-/- and Y2-/-) knockout mouse models and found no significant differences in osteogenesis between the two models [114]. This suggests that the effects of Y2R deletion on bone are not amplified. Additionally, Baldock et al. observed a downregulation of Y1R in the bone tissue of Y2-/- mice, indicating that the Y2R-dependent signaling pathway has an additional action on bone homeostasis by regulating the expression of Y1R by osteoblasts. Furthermore, the rapid increase in bone mass in adult mice after central deletion of Y2R makes Y2R a promising candidate for monitoring and/or new treatments for osteoporosis.

As previously mentioned for leptin, NPY levels have also been found to be altered in various neurodegenerative diseases, including AD and PD. Numerous studies have demonstrated alterations in NPY levels in both plasma and specific brain regions in AD, suggesting a potential role for NPY in the disease’s pathogenesis. For example, a transgenic mouse model of presymptomatic AD revealed a significant reduction in hippocampal NPY cells, accompanied by early impairment of neuronal network activity [115]. Furthermore, NPY mRNA expression was found to be decreased in both hippocampal and cortical regions of the same transgenic mouse model [116]. In the brains of AD patients, hippocampal NPY levels were significantly reduced, and this reduction was associated with an altered number of NPY receptors [117,118]. Additionally, a correlation was observed between plasma and cerebrospinal fluid NPY levels and the disease’s severity, with both levels decreasing as the disease progressed [119,120]. Interestingly, several in vivo and in vitro studies have demonstrated that NPY exhibits neuroprotective effects in AD. For instance, administering C-terminal fragments of NPY into the brains of transgenic mice with amyloid precursor protein improved neurodegenerative pathology [121]. Moreover, C-terminal amide fragments of NPY protected human neuronal cells from the neurotoxic effects of Ab. In a mouse model of AD with amyloid-b, a single intracerebroventricular administration of NPY prevented depressive-like behavior, spatial memory impairment, and oxidative stress following amyloid-b administration in mice [122].

Based on the recent discussion, it is evident that NPY can function as an anti-apoptotic, anti-inflammatory, and pro-phagocytic agent. Consequently, it holds the potential to effectively halt or slow disease progression. However, given NPY’s multifaceted physiological functions, its clinical application presents several critical considerations. In this context, the development of specific agonists or antagonists targeting NPY receptors becomes paramount in the clinical setting. In recent years, several agonists and antagonists against Y receptors have been developed and tested as research tools and in clinical trials [123]. Nevertheless, these drugs have yet to be translated into clinical applications. Another significant aspect to consider is the mode of administration of NPY as a therapeutic agent. NPY exhibits poor ability to cross the BBB, low oral bioavailability, and a short half-life. While previous studies have demonstrated the feasibility of intranasal administration of NPY in rodents [124] and humans [125], further research is needed to explore its potential in clinical settings.

### 4.3. Semaphorins

Semaphorins are a family of cell surface and soluble proteins that play a crucial role in regulating cell–cell interactions, cell differentiation, and various cellular functions.

Semaphorins are classified into eight distinct classes (C1–C8), with classes C3 through C7 being exclusively associated with vertebrates. Their actions are made possible by binding to specific receptors, known as plexins, which are further categorized into four groups: plexins A, B, C, and D.

Semaphorins and their receptors were initially discovered in the nervous system, although these molecules are also expressed in other tissues [126]. The semaphorin–plexin system is involved in various biological processes, including oncogenesis, angiogenesis, and immune responses. Numerous studies have demonstrated that the semaphorin–plexin system plays a crucial role in communication between osteoclasts and osteoblasts [127]. Semaphorins directly implicated in bone metabolism include semaphorins 3A, 4D, and 7A (Sema3A, Sema4D, and Sema 7A).

Sema3A primarily functions in the growth and development of vascular and nervous tissues. However, research conducted in animal models has demonstrated that Sema3A-knockout mice exhibit severe abnormalities not only in the nervous and cardiovascular systems but also, and particularly, in the skeletal system [128]. Given the extensive innervation and vascularization of bone, the development of the skeletal system exhibits significant similarities with the cardiovascular and nervous systems, particularly in the areas of cell differentiation and migration. Consequently, it is unsurprising that Sema3A plays a role in bone metabolism. Additional research has reinforced the dual function of Sema3A as a regulator of both osteoblast and osteoclast activity. It simultaneously exerts both stimulatory and inhibitory effects on bone formation and resorption, acting as a positive regulator in osteoblastogenesis (enhancing Wnt-induced signaling) and as a negative regulator in osteoclast differentiation [127,129].

Sema3A plays a pivotal role in bone remodeling processes, not only by influencing the cellular components involved but also by impacting the development of sensory nerves. Fukuda and colleagues demonstrated that neuron-specific Sema3A-deficient mice exhibit diminished bone mass due to an imbalance in bone remodeling, favoring resorption over formation [3]. Interestingly, Sema3A supplementation has been demonstrated to augment callus volume and BMD in osteoporotic rats at four weeks post-fracture. This stimulation further induces ossification and callus remodeling at eight weeks post-fracture [130]. These findings suggest the potential of Sema3A in regulating bone turnover.

Sema4D is a membrane semaphorin exclusively expressed by osteoclasts. It exhibits strong similarities with integrin, another protein commonly found in osteoclasts [131]. During bone resorption processes, Sema4D exerts an inhibitory effect on osteoblasts while simultaneously stimulating osteoclast formation and activity. Interestingly, Sema4D-knockout mice have been observed to exhibit increased bone mass, likely due to accelerated rates of bone formation. A similar phenotype was observed in mice lacking Plexin-B1, suggesting that Plexin-B1 acts as a functional receptor for Sema4D [132,133].

Sema7A plays a pivotal role in bone remodeling, particularly in the differentiation of osteoclasts and osteoblasts. The supplementation of recombinant Sema7A has been demonstrated to enhance osteoblast migration through integrin β1, simultaneously increasing the formation of mature osteoclasts [134] (Delorme et al., 2005). This suggests that Sema7A functions as a stimulator of osteoclast and osteoblast migration during bone remodeling. Additionally, Sema7A polymorphisms have been associated with an increased incidence of fractures and a decreased BMD in postmenopausal women [135], indicating a potential role for Sema7A in maintaining bone homeostasis.

### 4.4. Dopamine

Dopamine (DA), a neurotransmitter synthesized in both the CNS and the periphery, exerts its actions by binding to G protein-coupled receptors (GPCRs). Widely expressed throughout the body, DA receptors (Drds) function in both the peripheral and CNS. All Drds are metabotropic, leading to the formation of second messengers that trigger or block specific cell signaling pathways [136].

Dopaminergic signaling pathways are essential for maintaining physiological processes. An imbalance in this system can lead to dysfunctions associated with neurodegenerative diseases. The dopaminergic system plays several crucial roles in neuromodulation, including movement and motor control [137], spatial memory function [138], and cognitive function [137].

DA, beyond its significance in the CNS, plays a crucial role in the skeletal system. At the bone level, DA interacts with specific receptors on the cell surface, influencing bone cell proliferation, differentiation, and function [139].

During bone formation, DA plays a crucial role by enhancing the activity of osteoblasts. This stimulation accelerates the synthesis and mineralization of the bone matrix. Conversely, DA can also inhibit osteoclast activity, which is responsible for bone resorption. By reducing bone resorption, DA helps maintain a balanced bone mass [140].

DA also indirectly influences bone metabolism by regulating the secretion of hormones such as PTH and vitamin D hormone [141].

At the level of the CNS, DA primarily regulates motor function in PD. However, the degeneration and death of dopaminergic neurons in the substantia nigra lead to a drastic decrease in DA levels, resulting in motor disorders [142].

Observational and longitudinal studies have shown that PD patients are at a higher risk of osteoporosis and low BMD compared to controls [143].

A recent study conducted by Handa et al. revealed that Drds are expressed in osteoblasts (Drd3 and Drd4) and osteoclasts (Drd1 and Drd3), and they play a crucial role in maintaining bone homeostasis. Furthermore, the study demonstrated an increased bone resorption and a suppressed bone formation in an in vivo model of neurodegeneration of dopaminergic neurons [144]. Therefore, it can be inferred that degeneration of dopaminergic neurons in PD patients not only has purely neurological consequences but also bone consequences due to the disruption of the delicate balance between bone formation and resorption.

Recent studies conducted in mouse models have indicated that the effects of DA on the osteogenic differentiation of BMSCs may be influenced by the dose of DA administered [139]. These findings suggest that DA could potentially be a therapeutic agent for treating conditions such as osteoporosis and inflammatory bone loss.

### 4.5. Serotonin

Serotonin (5-HT), a neurotransmitter derived from tryptophan metabolism, is produced both in the CNS and peripherally, particularly in the gastrointestinal tract and platelets [145,146]. Peripheral 5-HT accounts for the majority of 5-HT produced in the body (~95%), while the brain contributes only a smaller fraction [147,148]. As a neurotransmitter, 5-HT regulates various functions, including mood, reward, anger, perception, appetite, aggression, attention, memory, and sexual desire. At the peripheral level, 5-HT plays a crucial role in regulating major organ functions, such as glucose homeostasis and lipid metabolism [146,149].

Interestingly, 5-HT also exerts significant influence on the skeletal system, particularly at the level of bone metabolism.

These effects are triggered by the binding of 5-HT to its specific receptors present in major bone cell types, including osteoblasts, osteocytes, and osteoclasts [150].

As reported by Bliziotes [151], 5-HT produced as a neurotransmitter acts centrally to inhibit bone resorption and promote bone formation.

The influence of 5-HT on bone tissue modulation within the CNS is intricately linked to leptin and the sympathetic nervous system. Central 5-HT exerts inhibitory effects on sympathetic nervous system activity, thereby indirectly preventing bone resorption and facilitating bone growth. Conversely, leptin can induce suppression of central 5-HT, concomitantly increasing sympathetic activity and adversely affecting bone formation. Notably, selective 5-HT reuptake inhibitors have been associated with diminished bone density and heightened fracture susceptibility [152].

Conversely, at the peripheral level, 5-HT directly inhibits bone formation [147,148].

These studies suggest that elevated peripheral 5-HT levels can lead to structural damage and decreased bone density. 5-HT is thought to directly inhibit bone formation and promote bone resorption through receptors present in bone tissue, thereby hindering the growth and differentiation of osteoblasts [150].

The close interconnection between 5-HT functions at CNS and bone levels is evident from the growing clinical evidence regarding side effects, such as a decrease in BMD and an increased risk of fractures, from the use of antipsychotics at the bone level in patients undergoing this therapy, particularly those with schizophrenia.

Bone cells possess dopaminergic, 5-HT, and adrenergic receptors that, when activated by binding to their specific ligands, initiate various pathways that regulate bone deposition and resorption processes. DA has been demonstrated to directly inhibit osteoclastogenesis through the D2/cAMP/PKA/CREB pathway [140]. Conversely, DA itself stimulates osteoblastogenesis through the D1/ERK 1 and 2/Runx2 pathway [153]. As previously mentioned, centrally produced 5-HT reduces bone resorption and enhances bone formation. However, peripherally, 5-HT directly acts on osteoblasts to inhibit bone formation [147].

Data suggests that 5HT2B and 5HT6 receptors play a crucial role in maintaining bone mass. It has been proposed that bone turnover is regulated by the adrenergic receptors Adrb2 and Adra1, which influence RANKL and OPG levels [154]. Additionally, activation of Adrb2 inhibits the phosphorylation of CREB, leading to decreased osteoblast proliferation. Furthermore, Adrb2 can activate the cAMP/PKA/AP1 pathways, which regulate bone formation [155].

Since antipsychotics primarily target DA and 5-HT receptors, as well as, to a lesser extent, adrenergic receptors, it is reasonable to speculate that antipsychotics may disrupt bone formation and resorption by acting on the cAMP/PKA/CREB, ERK 1/2, AP1, and Jab1/Smad1, 5, 8/BMP2 pathways.

These observations raise several critical issues regarding the use of certain antidepressants. A comprehensive meta-analysis conducted on fourteen observational studies involving over one and a quarter million participants provided substantial and consistent evidence of the detrimental impact of novel-generation antidepressants, particularly the widely utilized selective 5-HT reuptake inhibitors (SSRIs), on bone health. The analysis revealed statistically significant correlations with a decrease in BMD at all examined sites and a doubling of the risk of hip fractures, which are associated with significant morbidity. These findings raise serious concerns about the skeletal safety of chronic use of these medications. Health professionals must meticulously consider the risks of adverse bone effects and fractures when balancing the benefits of psychiatric treatment against the potential side effects of antidepressant therapy for individual patients. Close monitoring of bone health and preventive strategies to preserve skeletal integrity may be necessary for patients requiring long-term antidepressant treatment. Further research is urgently required to ascertain the optimal treatment approaches that can sustain bone strength and minimize the risk of fractures in the numerous patients currently receiving novel antidepressants [156].

### 4.6. Norepinephrine

Norepinephrine (NE), a neurotransmitter belonging to the catecholamine family, is primarily synthesized by noradrenergic neurons in the locus coeruleus (LC), a region of the brainstem. NE plays a crucial role in cognitive processes, particularly cognitive flexibility and active memory [157]. The synthesis of NE in neurons begins with the amino acid tyrosine, which is converted to DOPA and subsequently to DA. Finally, DA is converted into NE by the enzyme beta-hydroxylase [158]. Once synthesized, NE is released into the synaptic space, where it interacts with post- and presynaptic adrenergic receptors (ARs) to regulate neuronal and non-neuronal cell function.

The modulation of different molecular signaling pathways by NE can have both detrimental and neuroprotective effects, depending on various factors such as the concentration of NE, the subtype of adrenergic receptor stimulated, the duration of stimulation, and the specific stimuli.

NE also plays a significant role within the bone-brain axis. It exerts its action on bone metabolism and function by binding to a and β-ARs on the surface of bone cells [159,160].

Low NE concentrations primarily impact osteoblasts and osteoclasts by binding to a-ARs. Activation of a1-AR leads to the expression and release of RANKL from osteoblasts, ultimately triggering osteoclastogenesis [161]. The effects of NE binding to b-ARs on bone metabolism are contingent upon the specific subtype of receptors expressed in various skeletal cells. NE binding to b1-AR predominantly exhibits anabolic effects. On the one hand, it stimulates osteoblast activity, resulting in increased bone formation, particularly in the periosteum, through activation of the somatotropin-insulin-IGF-1 axis. On the other hand, it inhibits osteoclast activity and reduces bone resorption through the gonadotropin-releasing hormone-gonadal axis [162,163].

In contrast, binding to the b2-AR affects osteoblasts and osteocytes, leading to inhibition of bone formation. Additionally, NE stimulates the migration of BMSCs toward bone formation and osteogenesis. Furthermore, NE, through binding to the b-3AR receptor, regulates the differentiation of BMSCs by influencing the bone marrow microenvironment, contributing to bone formation and repair processes [164].

As further evidence of the brain–bone axis’s robustness and complexity and the role of its various components in related disorders, it is worth focusing on one aspect in particular: the active involvement of NE in PD. Scientific evidence increasingly suggests that the NE plays a crucial role in non-motor symptoms of PD, such as mood disorders, cognitive impairments, and sleep disturbances [165].

Moreover, noradrenergic neurons in certain brain regions may be affected by the pathological processes of PD, further intensifying patients’ symptoms. Dysfunction of the peripheral noradrenergic system in PD patients leads to an imbalance in bone metabolism, resulting in disruptions in bone formation and resorption processes [166].

Typically, PD patients experience a relative increase in bone resorption and a relative decrease in bone formation, leading to a decrease in BMD. This, in turn, significantly increases the risk of fractures, significantly impacting patients’ quality of life and prognosis [167]. Furthermore, abnormalities in the peripheral NE system may also affect the healing process after fractures in PD patients.

Numerous studies have demonstrated how alterations in NE can impact various aspects of fracture healing, including local inflammatory responses, cell proliferation and differentiation, and angiogenesis at the fracture site. For instance, NE impairments can lead to impaired functional coordination between osteoblasts and osteoclasts at the fracture site, preventing bone callus formation and remodeling. Consequently, fracture healing is delayed [168,169,170].

A comparable argument pertains to NE as to 5-HT. Specifically, there is substantial and consistent evidence demonstrating the detrimental impact of novel-generation antidepressants, particularly the extensively utilized selective serotonin-norepinephrine reuptake inhibitors (SNRIs), on both bone health and periodontal status [171].

In this context, a similar discussion is warranted regarding the widespread use of gabapeptide-based drugs, such as gabapentin (GBP). Initially developed for treating epileptic seizures, these drugs are now commonly used as an alternative to opioids for various chronic pain conditions. Their popularity has led them to become among the best-selling drugs globally. These drugs mimic the action of GABA, the primary inhibitory neurotransmitter in the cerebral cortex, by binding to the extracellular subunits of voltage-gated calcium channels (VSCCs). This binding reduces the magnitude of calcium currents [172].

While gabapeptinoids are undoubtedly beneficial for treating epileptic seizures, they can have adverse effects on tissues where VSCCs are present. Notably, studies have documented the negative impact of these antiepileptic drugs (AEDs) on bone health. Long-term use of AEDs is associated with detrimental bone health and an increased risk of osteoporosis [173]. A 2019 study [174] demonstrated that GBP inhibits both osteoclastogenesis and osteoblastogenesis. This inhibition leads to a significant increase in osteoclast apoptosis and a corresponding decrease in osteoclast- and osteoblast-specific gene expression. Consequently, bone quantity (BMD) and quality (lower bone strength and higher risk of fragility) are reduced, increasing the likelihood of bone fractures due to increased fragility.

It can therefore be concluded, as previously mentioned in relation to antidepressants, that it is crucial to adopt a well-considered approach to the use of these drugs to preserve skeletal integrity as effectively as possible.

### 4.7. Estrogens

Estrogens play an extremely significant role in the processes of skeletal growth and maintenance of bone mass in both males and females. These hormones interact with their specific ER α and ER β receptors (ERs) at the growth plate and mineralized bone levels from early human development to adulthood [175]. During puberty, estrogen begins to play its role in the pubertal growth phase by binding to ERs on osteoblasts and chondrocytes. This binding stimulates and amplifies osteoblastic activity while inhibiting osteoclastic activity [175,176]. After menopause, women experience a decline in the levels of these hormones, leading to accelerated bone loss and an increased risk of osteoporosis [177].

Estrogens, potent neuroprotective agents, play a crucial role in neuronal development in the adult brain. By binding to their receptors, estrogens actively participate in the processes of differentiation, proliferation, and protection against inflammatory processes in neurons, particularly dopaminergic neurons [178].

Neurodegenerative diseases, particularly AD, are associated with estrogen depletion. Studies have shown that women are more prone to developing AD and experiencing a faster decline in cognitive function compared to men [179]. Furthermore, a meta-analysis has suggested that estrogen replacement therapy might be beneficial for postmenopausal women with neurodegenerative diseases [180]. Numerous studies have demonstrated the neuroprotective role of estrogens, which include their antioxidant properties, DNA repair capabilities, induction of growth factor expression, and regulation of cerebral blood flow. PD primarily affects motor skills, leading to cognitive impairment. Interestingly, women with lower estrogen levels are more susceptible to developing PD compared to those with higher estrogen levels [180,181]. This suggests that the estrogen receptor (ER) may have neuroprotective effects against PD.

### 4.8. Parathormone

Parathormone (PTH), synthesized by parathyroid glands, plays a pivotal role in regulating calcium-phosphorus metabolism and bone metabolism.

Parathyroid cells perceive minute variations in extracellular calcium levels via the calcium-sensitive receptor (CaSR). This signaling pathway induces an elevation in intracellular calcium and suppresses the secretion of parathyroid hormone (PTH). Consequently, PTH is excreted in response to diminished serum ionized calcium (Ca^2+^) levels [182]. However, elevated Ca^2+^ inhibits PTH gene transcription and stability [183,184].

The action of parathyroid hormone (PTH) on bone is mediated by its binding to the specific PTH1R receptor, a member of the GPCR family.

PTH stimulates bone resorption by acting on the RRO pathway. Specifically, PTH modulates the expression of RANKL and OPG in osteoblast precursors and in osteocytes. RANKL binds to RANK on the surface of hematopoietic precursors of osteoclasts and mature osteoclasts, promoting their differentiation, survival, and activity; OPG binds to RANKL, inhibiting RANK-RANKL interaction [185,186].

Studies in mouse models have demonstrated that continuous PTH infusion leads to an increase in mRNA encoding for RANKL and a decrease in mRNA encoding for OPG. Consequently, the RANKL/OPG ratio rises, which in turn stimulates osteoclastogenesis and bone resorption [187].

Similarly, in humans, the RRO pathway is also regulated by PTH through a similar mechanism. Individuals with primary hyperparathyroidism exhibit elevated levels of both RANKL and OPG and the RANKL/OPG ratio compared to healthy controls [188]. These findings suggest increased rates of bone resorption [189].

Prolonged exposure to elevated levels of PTH induces bone remodeling and demineralization, leading to a reduction in BMD and an increased risk of all osteoporotic fractures [190].

Although PTH is widely recognized as the primary regulator of calcium and bone metabolism, it is essential to recognize the existence of a family of PTH-related hormones with a wide range of potential sites and tissues of action. Mammals possess several PTH-related peptides, including PTH, PTH-like hormones (PTHrP and PTHLH), and others.

The presence of PTH in the cerebrospinal fluid (CSF) is now firmly established, and there is a strong correlation between alterations in CSF PTH levels and the incidence of neurological disorders [191]. Similarly to PTH, PTHrP has been detected in various regions of the CNS, including the hippocampus, hypothalamus, pituitary, and cortex, and has been measured in the CSF [192]. In vitro studies suggest that PTHrP may play a protective role in neurons against prolonged depolarization toxicity, commonly referred to as “excitotoxicity” [193].

Furthermore, PTHrP is expressed in glia and astrocytes, where it appears to be involved in inflammatory responses to brain injury in animal models [194]. Additional evidence indicates its potential role in modulating nerve regeneration [195] and cerebral vasculature [196].

The distribution of PTH receptors in the CNS is also well-known. In humans, PTH1R is expressed in various regions, including the hippocampus, amygdala, hypothalamus, caudate nucleus, corpus callosum, subthalamic nucleus, thalamus, substantia nigra, and cerebellar astrocytes.

It is unsurprising, therefore, that clinical evidence supports the idea that altered levels of PTH and PTH-related peptides exert an impact on the CNS. Patients diagnosed with primary hyperparathyroidism and hypoparathyroidism exhibit neuropsychological and cognitive manifestations, resulting in a diminished quality of life. The precise mechanism underlying these effects remains elusive. It is currently unclear whether the effects on the CNS can be attributed to a direct action of PTH on the brain or whether they are secondary to hypo- and hypercalcemia. Nevertheless, the extant evidence suggests that PTH and PTH-related peptides play a role in mediating at least some of these effects.

Certainly, the prominent feature in patients with hypoparathyroidism is the significant neuropsychological involvement, manifesting in neurological and cognitive symptoms such as depression, fatigue, anxiety, and memory dysfunction. The clinical evidence and available literature [197] unequivocally demonstrate the profound impact of parathyroid hormone-related peptides on the CNS.

Alterations in PTH levels have also been observed in patients with PD, which may be linked to abnormal bone metabolism. Some studies suggest that PTH might play a role in the development of PD. PTH can influence dopaminergic neurons by affecting various pathways, including calcium signaling and modulating inflammatory responses [198].

Consequently, the clinical implications of these findings emphasize the urgent need for further research, both in vitro and in vivo, to unravel the intricate underlying mechanisms that remain largely elusive.

### 4.9. Osteocalcin

OC, the primary non-collagen protein in bones, closely associates with bone metabolism, participating in the processes of bone formation and loss [199,200].

It constitutes up to 3% of the total protein in bones and is synthesized by mature osteoblasts through the vitamin D system’s induction. Comprising a polypeptide chain of approximately 49 amino acids, OC’s most distinctive feature lies in the presence of three glutamic acid residues that undergo carboxylation by a vitamin K-dependent process. In its carboxylated form, OC exhibits a strong affinity for calcium ions and hydroxyapatite, the primary mineral component of the bone matrix. Consequently, it plays a pivotal role in the mineralization process, stabilizing and facilitating the formation of mineralized bone tissue. Additionally, OC is involved in the regulation of phosphate-calcium metabolism, which is essential for maintaining skeletal health [201].

The OC functions are regulated by its receptors. Currently, three receptors for OC have been identified in mammals: GPR37, GPR158, and GPRC6A, all of which are classified as GPCRs [202]. These receptors exhibit distinct regional distributions and perform diverse functions within the organism.

Depending on the availability of vitamin K, two types of OC are produced: carboxylated, which is involved in osteogenesis, and uncarboxylated, which can enter the blood circulation. Decarboxylation of OC results in the production of decarboxylated OC, which can also enter the circulation and participate in acidic bone resorption by osteoclasts.

There is now evidence suggesting that uncarboxylated OC (ucOC) can cross the BBB and bind to neurons, thereby exerting a regulatory function in the CNS [203]. Studies conducted in cellular and animal models have demonstrated that OC can promote neurite outgrowth. Additionally, maternal OC has been shown to cross the placental barrier, influence neurogenesis in the embryo, and play a neuroprotective role [6,204]. Furthermore, maternal OC has been found to affect learning and memory in adult offspring, regulating a wide range of neuronal activities associated with cognitive function and anxiety, including neurotransmitter synthesis and release, synaptic plasticity, brain-derived neurotrophic factor synthesis, neurogenesis, and autophagy [204,205,206].

OC, a protein crucial for bone health, has emerged as a key player in neurological disorders. A study on mice lacking OC revealed alterations in spatial learning, memory, and increased anxious and depressive behaviors [6]. Beyond cognitive function, OC plays a vital role in regulating mood and stress responses. It has been shown that ucOC stimulates the synthesis of monoamine neurotransmitters, including 5-HT, DA, and NE, which are essential for emotional state regulation [207]. Studies in animal models have demonstrated that OC deficiency leads to heightened anxiety and depression, which can be alleviated by external ucOC administration. Clinical studies further support the significance of OC in brain function. Serum levels of ucOC have been positively correlated with cognitive performance and memory in elderly individuals, while reduced levels have been observed in patients with AD and depression [208].

In this regard, it is worth mentioning the work of Shan, who conducted research on an AD mouse model. Their findings revealed that OC can alleviate cognitive impairment in AD mouse models by reducing the amyloid β burden and enhancing glycolysis in neuroglia [209]. In recent years, several studies in animal models have shed light on the significance of the OCN/GPR37 signaling pathway in regulating myelin homeostasis and the consequences of its dysfunction. For instance, Wu and colleagues demonstrated that in a mouse model of AD, there is an increase in myelin thickness, like what’s observed in OCN knockout mice [210]. Conversely, another study conducted in a mouse model of AD showed that inducing increased myelin turnover leads to a regression of cognitive dysfunction [211].

This evidence suggests that OC might be considered a potential biomarker of cognitive decline and neuropsychiatric disorders, and it raises the possibility of its therapeutic role in CNS disorders.

## 5. Other Regulatory Mechanisms

### 5.1. RhoA/ROCK

Ras homolog gene family member A (RhoA) is a small GTPase protein in the Rho family.

RhoA protein is expressed in various tissues, including normal human tissues, embryonic tissues, and stem cells. It primarily localizes in the plasma membrane, cytoplasm, and near cell–cell contacts and cell projections. RhoA plays a crucial role in multiple cellular processes, such as cell growth, transformation, and cytoskeleton regulation [212,213,214]. Rho-associated protein kinase (ROCK) exists in two isoforms, ROCK1 and ROCK2. These isoforms function as downstream effectors of Rho, exerting diverse biological effects. The RhoA/ROCK signaling pathway is a crucial signal transduction system that plays a pivotal role in cell growth, differentiation, migration, and development [215,216], including in the brain and bone [217]. Rho GTPases, which play fundamental roles in all cell types, are particularly important in the CNS and in regulating osteoclastic and osteoblastic activity. They play a pivotal role in neural development, influencing key processes like neuronal migration, differentiation, and proliferation [218,219]. Rac1, one of the Rho proteins with GTPase activity, plays a crucial role in promoting axon and dendrite survival, growth, and branching. It also contributes to the formation and maintenance of dendritic spines, which are the primary postsynaptic sites of excitatory synapses [220]. In contrast, RhoA exerts an inhibitory effect on these processes, leading to neuronal death, axonal and dendritic retraction, and the loss of spines and synapses [220,221]. Rac1 and RhoA are also pivotal regulators of neuronal migration in the developing CNS, with Rac1 generally promoting migration and RhoA inhibiting it [222]. Furthermore, the proper functioning of Rho GTPase signaling is essential for neuronal survival and the maintenance of neuronal architecture in the adult brain [220,223]. Therefore, it is a finely regulated balance that can be disrupted in the case of CNS disease and injury [224].

Alterations in Rho GTPase signaling have been implicated in a wide range of neurodevelopmental, neuropsychiatric, and neurodegenerative disorders, including intellectual disabilities, autism spectrum disorders, schizophrenia, depression, amyotrophic lateral sclerosis (ALS), PD, and AD [220,225].

Osteoclast cytoskeletal architecture and bone resorption activity must be precisely regulated for optimal bone remodeling. Disruption of this process is closely linked to osteoporosis. At the dawn of this century, it became evident that the GTPase protein RhoA plays a pivotal role in osteoclast development, mobility, and function [226]. Moreover, substantial evidence supports the significance of the RhoA/ROCK pathway in osteoblast differentiation and activation processes. Abnormalities in this pathway, which are common in neurodegenerative diseases as previously mentioned, can hinder osteoblast differentiation and activity, leading to reduced bone formation. Conversely, they may enhance osteoclast activity and increase bone resorption [227].

### 5.2. Notch

**Figure 5:**
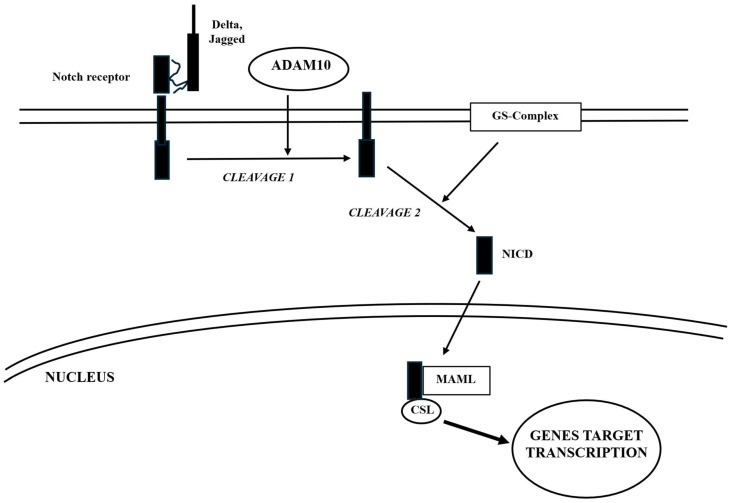
Notch signaling pathway. Notch ligands (Delta and Jagged) bind to Notch receptors, initiating their activation. This activation triggers two cleavages, catalyzed by ADAM-10 and the GS-Complex, leading to the release of NICD into the cytoplasm. NICD moves to the nucleus, where it forms a complex with CSL and MAML. This complex then activates the transcription of Notch target genes. GS-Complex: membrane-bound γ-secretase complex; NICD: Notch intracellular cytoplasmic domain; CSL: CBF1/Su(H)/Lag-1); MAML: mastermind-like coactivator.

The Notch signaling pathway, an evolutionarily conserved mechanism, controls various functions of stem cells, including cell fate determination, differentiation and proliferation regulation, and apoptosis induction [228]. Activated by specific ligands like Delta and Jagged, which bind to Notch receptors on the cell surface, the Notch pathway initiates a cascade of intracellular events that modulate the expression of target genes [229] (Figure 5).

Notch receptors, multifunctional transmembrane proteins, regulate cell differentiation, development, proliferation, and survival in various environments. Humans possess four Notch receptors (Notch1–4) and five ligands (δ-like 1, 3, 4, and Jagged-1, -2). This pathway plays a crucial role in the brain, regulating processes such as neurogenesis, cellular differentiation, and maintaining homeostasis within the nervous system [230].

The Notch signaling pathway plays an equally crucial role in bone development and homeostasis. Bone cells primarily express Notch1 and Notch2, while Notch3 is less expressed [231]. Therefore, it is an extremely important signaling pathway whose dysregulation may underlie various pathologies.

**Figure 6:**
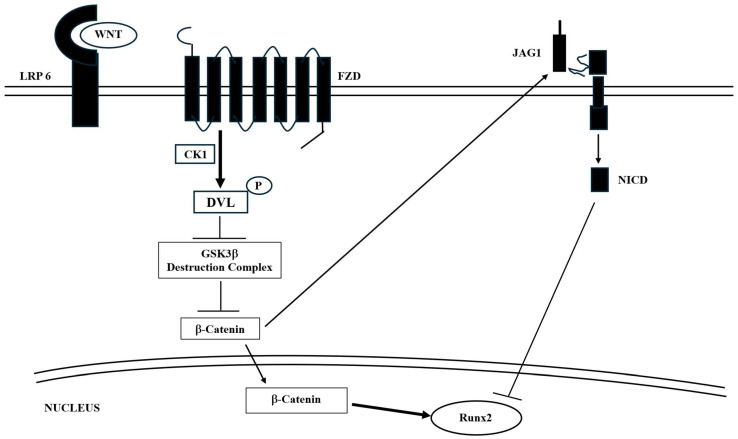
Notch in osteoblast differentiation. Osteogenic differentiation is initiated by the activation of Wnt/β-catenin. β-catenin, in turn, activates the Notch ligand JAG1, leading to a Wnt/β-catenin pathway inhibition by Hey1, a downstream target gene of Notch, that inhibits the transcriptional activity of Runx2 (essential for osteoblast proliferation, differentiation, and maturation), serving as a negative feedback mechanism. FZD: frizzled proteins; CK1: casein kinase 1; DVL: disheveled proteins.

As previously mentioned, the Notch signaling pathway plays a crucial role in maintaining a delicate balance between differentiation and the preservation of neural stem cells (NSCs). This balance is essential for the proper functioning of the CNS [232]. Disruptions in this pathway can lead to various neurodegenerative diseases, such as AD, multiple sclerosis, and ALS [233] (Figure 6).

On the bone level, the activation of Notch in osteoblasts, osteocytes, and stromal cells leads to a decreased ratio of RANKL to OPG. This inhibition of M-CSF gene expression further diminishes their capacity to support osteoclastogenesis and bone resorption. Consequently, a deficiency in Notch1 indirectly promotes osteoclastogenesis by augmenting the ability of osteoblast lineage cells to increase the RANKL/OPG expression ratio [231,234].

Notch signaling exerts a bidirectional influence on osteoblast differentiation, regulating its progression based on the stage and the timing of Notch activation [235]. While some studies indicate that Notch signaling activation can stimulate the mineralization process of osteoblasts [236,237], others demonstrate that overexpression of Notch1 can impede their differentiation by inhibiting the Wnt/β-catenin signaling pathway [238]. Notably, Notch1 not only hinders the terminal maturation of osteoblasts but also facilitates the formation of immature osteoblasts [239].

This complex interaction must be viewed as a whole and considered as a mechanism of mutual interaction and regulation during osteogenesis and bone development, with positive or negative outcomes depending on the different stages of cell maturation. Osteogenic differentiation is initiated by the activation of Wnt/β-catenin, which simultaneously inhibits Notch signaling. Conversely, Notch overexpression hinders osteoblastogenesis by blocking the Wnt/β-catenin pathway. Interestingly, β-catenin activates the Notch ligand JAG1, suggesting that the Notch pathway operates downstream of the Wnt/β-catenin pathway, serving as a negative feedback mechanism (Figure 6). Notably, Wnt/β-catenin-induced bone formation is further enhanced by Notch signaling, which leads to the upregulation of OPG. Furthermore, it is crucial to recognize that the transcription of the Runx2 gene, which is essential for osteoblast proliferation, differentiation, and maturation, is triggered by the activation of the Wnt/β-catenin pathway. Additionally, Hey1, a downstream target gene of Notch, inhibits the transcriptional activity of Runx2 on osteoblastic genes (Figure 6).

In the subsequent phase of osteocyte differentiation, Notch plays a role in suppressing Wnt/β-catenin signaling. This suppression prevents the nuclear aggregation of β-catenin and directly represses phosphorylated Akt. This evidence demonstrates that Notch signals with Wnt/β-catenin in both positive and negative directions, depending on the specific cell phase.

Forkhead box O1 (Foxo1) protein plays a crucial role in inhibiting osteoblast production by interacting with the Notch signaling pathway. Foxo1 forms complexes with notch intracellular cytoplasmic domain (NICD) and Mastermind, a gene encoding a MEF2 transcriptional co-activator, thereby exerting an inhibitory effect on the expression of specific genes [240,241].

For instance, Foxo1 inhibits nuclear factor of activated T cells (NFAT) signaling in activated T cells. Given that NFATc1 and NFATc2, in turn, promote the production of both osteoblasts and osteoclasts, contributing to bone formation [240], it follows that through its interaction with Foxo1, Notch directly and indirectly inhibits osteoblast and osteoclast production [241].

Given the extreme significance of Notch signaling in osteoblast differentiation and maturation, it is unsurprising that Notch signaling pathway-dependent regulation may have profound implications for comprehending and treating diseases such as osteoporosis [242] and osteosarcoma.

Activation of Notch signaling can result in two distinct effects on bone homeostasis [243], depending on the cellular context and time. As previously discussed, overactive Notch signaling in osteoblast precursors inhibits their differentiation [238], leading to decreased bone formation and osteoporosis [244]. Conversely, inhibition of Notch2 signaling in osteoblasts enhances osteoblast activity, thereby promoting bone formation [245], making it a potential therapeutic approach for osteoporosis.

Osteoclastogenesis is stimulated by Notch through both direct and indirect pathways. For instance, the transcription of Natcl, a gene crucial for osteoclastogenesis, is triggered by interactions between Notch2ICD and NF-kB in osteoclast precursors [246]. Consequently, inactivating Notch2 could be a promising dual strategy to manage osteoporosis, effectively promoting bone mass formation while simultaneously preventing bone resorption.

Abnormalities in the Notch pathway, which lead to its non-regulation, are the underlying cause of numerous types of cancer. At the bone level, osteosarcoma is a clear example. It occurs due to abnormal activation of Notch signaling in osteoblastic cells. In fact, it has been observed that the expression of certain genes downstream of the Notch pathway (HEY and HEY2) and some components of the Notch pathway itself (NOTCH1, NOTCH2, JAG, and DLL) is significantly elevated in cell lines and primary tissues of human osteosarcoma [247,248]. Several studies in murine models have shown that tumor growth is significantly inhibited by treatment with Notch y-secretase inhibitors [249]. This highlights the role of Notch in the etiology of osteosarcoma and the importance of its proper and balanced regulation for bone homeostasis.

Given the current considerations, therapeutic approaches that target the Notch signaling system emerge as a promising avenue for drug development in the management of bone disorders.

miRNAs, small single-stranded RNA molecules, regulate gene expression by inhibiting protein synthesis or disrupting mRNA. Studies have associated miRNAs with signaling pathways in bone differentiation and development, hematopoietic cells, and several types of bone-related malignancies. miR-34c plays a crucial role in maintaining bone homeostasis by directly targeting components of the Notch signaling system to regulate osteoclasts [250,251]. More specifically, miR-34c inhibits osteoclast formation by inhibiting the function of JAG1 and Notch-1, while miR-107 inhibits Notch-2 protein expression [252]. miR-637 and the miR-23a-27a cluster also hinder osteoblast development, while miR-206 expression decreases during osteoblast differentiation. Additionally, miR-146 and miR-29b function as suppressors of osteoclastogenesis. miRNAs actively participate in the regulatory processes of both programmed cell death and cell proliferation in bone-related malignancies, often by triggering aberrant activation of the Notch signaling pathway [253,254]. The interaction between miRNAs and Notch signaling is pivotal for bone growth and homeostasis. Therefore, the development of therapies based on miRNAs as therapeutic agents or targets presents a significant challenge with substantial potential.

Another possible approach is the use of specific inhibitors capable of preventing the formation of the Notch intracellular domain (NICD), the primary driver of Notch activity, thereby blocking an aberrant activation of Notch signaling pathways.

Dilawar et al. [255] report on two novel potent inhibitors. One of them is LY3039478, a Notch inhibitor that reduced Notch signaling and its downstream biological effects. The other inhibitor, CB-103, is a pan-NOTCH inhibitor that is the first in its class to precisely and directly target the NOTCH transcription complex, thereby disrupting signaling.

### 5.3. TNF-α

TNF-α, a potent pro-inflammatory cytokine, is secreted by various cell types, particularly mononuclear phagocytes. It activates cytocidal functions, contributing significantly to host defense.

TNF-α plays a crucial role in the development of inflammatory disorders, including PD, which exhibits a prominent inflammatory component in both motor and nonmotor aspects.

As previously mentioned, patients with PD exhibit increased bone resorption and decreased bone formation, leading to a decrease in BMD. In this context, TNF-α inhibits the differentiation and function of osteoblasts, further contributing to the reduction in bone formation.

TNF-α directly increases RANKL expression in bone cells, which promotes osteoclast formation and bone resorption. Furthermore, TNF-α directly upregulates sclerostin expression through NF-kB signaling, contributing to bone loss [256].

Sclerostin, a glycoprotein mainly produced by osteocytes, inhibits the Wnt signaling pathway, which promotes osteoblastic differentiation [257]. Sclerostin also suppresses osteoblastogenesis and activity, leading to a reduction in bone formation [258]. Conversely, sclerostin deficiency has been linked to sclerosteosis and Van Buchem’s disease, which are characterized by elevated bone mass [259,260].

It is crucial to highlight that the chronic and systemic elevation of TNF-α levels, associated with obesity, diabetes, alcohol abuse, and advanced age, presents a substantial clinical challenge, particularly in the orthopedic domain.

For instance, menopause, characterized by a progressive decline in estrogen production, triggers an increased secretion of IL-12 and IL-18 by macrophages, which subsequently activates TNF-α-secreting T cells [261]. Consequently, this leads to a surge in serum TNF-α concentrations and, notably, osteoclastogenic activity. Ultimately, these factors contribute to the development of osteoporosis.

Analogous complications are observed in obesity, where the chronic inflammation inherent in the condition can stimulate osteoclastogenesis through the upregulation of the NF-kB (RANK)/RANK pathway [262]. This is accomplished by elevated levels of TNF-a secreted by excess adipose tissue [263,264]. Table 1 provides a concise overview of the main key players involved in the brain–bone axis.

## 6. Emerging Technologies and Future Directions

Orthopedic conditions have emerged as global health concerns, but the limited understanding of the underlying pathological processes at the cellular and molecular level has limited the development of comprehensive treatment options for these disorders. The advent of single-cell RNA sequencing (scRNA-seq) technology has improved the clinical and biomedical approach based on the detailed examination of cellular and molecular diversity. Recent research using single-cell RNA sequencing (scRNA-seq) has significantly advanced our understanding of both bone and brain health, revealing cellular diversity and molecular mechanisms that were previously inaccessible.

A comprehensive review outlined methods for isolating high-quality single cells from mineralized skeletal tissues. It emphasized the use of mechanical dissociation, enzymatic digestion, and cell sorting techniques like FACS and MACS. These advances have enabled deeper insights into diseases like osteoarthritis, rheumatoid arthritis, and disk degeneration, and have helped identify novel therapeutic targets [265].

Similarly, single-cell RNA sequencing (scRNA-seq) provided insight into neurodegenerative disease. Researchers have combined long- and short-read sequencing in single cells to uncover transcriptomic differences in Alzheimer’s disease, Parkinson’s disease, and dementia with Lewy bodies. This approach revealed previously unknown mRNA variants and cell-type-specific expression patterns, offering new avenues for diagnostics and treatment. The therapeutic implications of this revolutionary technique are multiple, ranging from biomarker discovery, (scRNA-seq helps identify predictive biomarkers for treatment response) to drug repurposing (by mapping gene expression profiles, existing drugs can be matched to new targets) and cell therapy optimization (understanding cell lineage and differentiation helps refine stem cell-based therapies) [266].

Significant progress has been made in the field of biomarker discovery, driven by technological innovations and a deeper understanding of the molecular pathways associated with various diseases. The improvement was based on recent progress in high-throughput methods such as genomics, proteomics, and metabolomics. These technologies enable researchers to analyze large datasets and identify potential biomarkers with high specificity and sensitivity.

Recent studies have highlighted the potential of liquid biopsies, which involve analyzing biological fluids such as blood or cerebrospinal fluid for biomarkers. This approach offers a less invasive alternative compared to traditional tissue biopsies, making it particularly advantageous for monitoring disease progression or treatment responses.

In neurodegenerative disease, the identification of neurofilament light chain (NfL) in blood has been discovered as a promising biomarker for several neurodegenerative conditions, including Alzheimer’s disease and amyotrophic lateral sclerosis (ALS), providing new insights into neuronal damage and anticipating the diagnosis [267].

In addition, advancements in imaging techniques, such as positron emission tomography (PET) and magnetic resonance imaging (MRI), have enhanced the ability to visualize pathological processes in vivo. These imaging modalities can be associated with biomarker analyses, establishing a more comprehensive understanding of disease mechanisms and progression.

This integrating multi-modal approach is based on combining biochemical assays with advanced imaging and increasing the reliability of biomarker identification, thereby representing a promising strategy for future research.

Moreover, artificial intelligence (AI) and machine learning are increasingly being applied to biomarker discovery. These computational tools can analyze vast amounts of data to uncover patterns that may not be readily apparent, leading to the identification of novel biomarkers that have significant diagnostic or prognostic value.

In summary, the integration of innovative technologies and interdisciplinary research would enhance the efficacy of biomarker application, ultimately leading to an improved patient outcome [268].

## 7. An Emblematic Condition: Dementia

Up to this point, we have investigated several players in the brain–bone axis by examining their mechanisms of action in physiological and pathological situations.

It therefore seems appropriate to conclude this review by considering a condition prevalent in many CNS diseases that directly involves the bone compartment, serving as a striking example of the profound interconnection between the two districts that constitute this axis: dementia.

Cognitive impairment, dementia, and fragility fractures are common among the elderly population, particularly those over the age of 70, in developing countries [269]. Fragility fractures, particularly in the spine and hip, significantly impact elderly women and men aged 65 and above [270]. Moreover, dementia, affecting one in ten older individuals, becomes more prevalent among those over 80 [271]. Approximately one-third of individuals with dementia experience falls or fractures that necessitate hospitalization, leading to a decline in physical activity [272]. This decline can further contribute to bone loss, malnutrition, and sarcopenia, ultimately increasing mortality [273]. Consequently, a detrimental cycle ensues, having substantial social and economic repercussions, particularly in developing nations.

Numerous studies have underscored the strong bidirectional connection between bone fragility, BMD, cognitive decline, and dementia [274,275]. Among the various pieces of evidence, it has been demonstrated that adult subjects with dementia, cognitive impairment [276], and AD [277] are at a higher risk of bone fragility fractures. In fact, research has demonstrated that individuals with dementia exhibit lower BMD compared to age-matched controls, leading to a heightened susceptibility to hip fractures. Notably, this susceptibility is more prevalent in men than in women [278].

Several studies have underscored the connection between bone fragility fractures and the subsequent onset of cognitive impairment and dementia. They have shown that elderly individuals with a history of bone fragility fractures are more prone to developing dementia compared to their age-matched counterparts who have not experienced such fractures [279,280]. In this context, it is worth mentioning the study conducted by Takahashi and colleagues [281], which revealed a significant rise in cognitive impairment among elderly individuals six months after a vertebral fracture. All this evidence has led to the well-founded hypothesis that fragility fractures may accelerate the neurodegenerative processes that ultimately lead to the onset of dementia.

Among the numerous studies on this topic, the work of Yaffe [282] stands out. This study revealed a significant correlation between osteoporosis, BMD, dementia, and cognitive impairment. The study, conducted on a female population with an average age of 75.8 years, demonstrated that cognitively intact women with osteoporosis were at a high risk of developing cognitive impairment within a 4–6 year period. Evidence from subsequent studies confirmed the association between low BMD, osteoporosis, and elevated risk of AD onset 5 years after the first assessment, regardless of lifestyle habits [283]. Similarly, a study conducted in 2018 [284] revealed that individuals with both AD and osteoporosis experience a more severe form of dementia compared to those without osteoporosis.

Another study [285] conducted on a healthy population over 50 years old revealed that the association between BMD and cognitive impairment is more pronounced in women compared to men. This suggests that low BMD and osteoporosis may serve as surrogate markers for estrogen exposure throughout life, particularly in individuals with early AD. Furthermore, a 2021 study [286] indicated that low BMD is correlated with alterations in white matter and lower cognitive performance, independent of canonical cerebrovascular risk factors.

In other words, alterations in brain function and structure in early AD subjects, but not in non-demented controls, may contribute to bone loss through a regulatory pathway that influences bone remodeling.

It is crucial to recognize that specialized cells in the brain and bone system share several signaling and pathogenic mechanisms [287]. Among these, neuropeptides (NPs) and osteokines (OKs) stand out. NPs mediate communication between the brain and the periphery, while OKs facilitate communication between the bone and the brain. Both NPs and OKs play a role in regulating body metabolism [288,289,290].

Over the years, numerous publications have delved into the study of specific genes involved in dementia, such as apolipoprotein E4 and amyloid precursor protein (APP), and their role in maintaining bone homeostasis [291]. Notably, bone-derived APP has been observed to enhance osteoblast survival and bone formation by reducing the production of mitochondrial reactive oxygen species (ROS) [292]. Additionally, protein kinase B (PKB/AKT) has been shown to affect superoxide overproduction, contributing to the onset of bone and brain diseases [22,293,294].

## 8. Conclusions and Future Perspectives

The brain–bone axis, a subject of growing research, has unveiled the intricate bidirectional communication between the CNS and skeletal metabolism. This review delves into the complex mechanisms that regulate and characterize this axis, its key players, and the clinical implications of its malfunctions in various pathological situations involving the skeletal and CNS. We particularly highlight the role of neural pathways, hypothalamic neuropeptides, neurotransmitters, and bone-derived factors in regulating bone metabolism and brain function.

We subsequently focused on the therapeutic prospects and practical realities of numerous of these regulators. Some are already targets (RANKL with dmab) or clinical treatment agents (such as leptin and NPY). Others are targets of treatments and therapies in the preclinical trial phase (TREM2 and NPY with specific antibodies or antagonists). Still others (such as DA and NE) may even be “collateral targets” in the utilization of antidepressants, underscoring the necessity of a comprehensive and rigorous assessment that harmonizes the benefits of psychiatric treatment with the potential adverse effects of antidepressant therapy for individual patients. Finally, there are others, such as Notch, with exceptionally promising potential.

Therefore, the in-depth study of this axis has expanded our understanding of the pathophysiological mechanisms of skeletal and neurological disorders, opening potential avenues for developing diagnostic tools, therapeutic interventions, and preventive strategies that target both skeletal and neurological health. However, many unresolved issues remain, necessitating further research to fully comprehend the intricate complexity of this axis.

## Figures and Tables

**Table 1:** Summary overview of main key players in the brain–bone axis and related diseases.

Brain–Bone Axis Main Players	Origin	Action	Pathology/ Condition
Wnt-β-catenin pathway	Ubiquitous	- Promotes osteoblast differentiation, proliferation, and mineralization. - Induces OPG expression to prevent bone resorption.	Osteoporosis; AD
RANKL/RANK/OPG system	T cells/bone cells/osteoblasts, bone marrow stromal cells, B cells, and dendritic cells	- Pivotal role in regulating bone remodeling by balancing bone formation and resorption. - Crucial role in immune and related processes regulation. - Critical role in modulating neuroinflammatory processes.	Rheumatoid arthritis; osteoporosis; AD
TREM2	Single immunoglobulin variable (IgV) domain receptor family	- Pivotal role in maintaining CNS tissue homeostasis. - Crucial role, in conjunction with the Wnt pathway, in regulating β-catenin signaling to promote osteoblast differentiation and proliferation.	AD; PLOSL
FGF-23	Bone	- Regulation of bone remodeling by influencing the differentiation of osteoblasts and osteocytes. - Plays a pivotal role in promoting neurogenesis, which is essential for preserving cognitive functions.	Cognitive and memory decline; bone remodeling alterations
Leptin	Adipocytes	- Pivotal role in bone metabolism by direct and indirect action. Direct action: stimulates the proliferation of osteoblasts while simultaneously inhibiting the activity of osteoclasts. Indirect action: regulation of the hypothalamic–pituitary–adrenal (HPA) and growth hormone/insulin-like growth factor-1 (GH/IGF-1) axes, both of which are essential for maintaining bone homeostasis. - Neuroprotective action on the CNS.	Obesity; AD; anorexia nervosa; hypothalamic amenorrhea
Neuropeptide Y (NPY)	Hypothalamus (arcuate nucleus, ARC)	-Neural mediator in bone remodeling processes and the maintenance of bone homeostasis.	AD; PD
Semaphorins (Sem)	Ubiquitous	- Regulators of various biological processes such as angiogenesis, immune responses, and bone metabolism. - In particular, in bone: #Sem3A: Regulator of both osteoblast and osteoclast activity, acting as a positive regulator in osteoblastogenesis (enhancing Wnt-induced signaling) and as a negative regulator in osteoclast differentiation. #Sema4D: Exerts an inhibitory effect on osteoblasts while simultaneously stimulating osteoclast formation and activity. #Sema7A: Stimulator of osteoclast and osteoblast migration during bone remodeling.	Cancer; autoimmune diseases (like rheumatoid arthritis and multiple sclerosis); metabolic disorders
Dopamine	Hypothalamus and adrenal glands (in smaller percentage)	- Crucial roles in neuromodulation, including movement and motor control, spatial memory function, and cognitive function. - Pivotal role in maintaining bone homeostasis, directly by enhancing the activity of osteoblasts and inhibiting osteoclast activity; indirectly by regulating the secretion of hormones such as PTH and vitamin D.	Osteoporosis; cognitive decline; PD
Serotonin (5-HT)	CNS (by serotonergic neurons); gastrointestinal tract and platelets (peripheric production)	- 5-HT produced as a neurotransmitter acts centrally to regulate various functions, including mood, reward, anger, perception, aggression, attention, and memory. At the peripheral level, 5-HT plays a crucial role in regulating major organ functions, such as glucose homeostasis and lipid metabolism. - In bone, 5-HT produced as a neurotransmitter acts centrally to inhibit bone resorption and promote bone formation. Conversely, at the peripheral level, 5-HT directly inhibits bone formation.	Depression; osteoporosis; skeletal integrity loss associated with long-term antidepressive treatment
Norepinephrine (NE)	Locus ceruleus	- Crucial role in cognitive processes, particularly cognitive flexibility and active memory. - Significant action on bone metabolism and function by binding to α- and β-ARs on the surface of bone cells. Binding to α1-AR leads to the expression and release of RANKL triggering osteoclastogenesis. Binding to β1-AR has anabolic effects, stimulating osteoblast activity and inhibiting osteoclast activity. Binding to the β2-AR has catabolic effects, leading to inhibition of bone formation.	Cognitive impairments; sleep disturbances; PD; bone fragility and risk fracture; skeletal integrity loss associated with long-term antidepressive treatment
Estrogens	Ovaries, corpus luteum, placenta and adipose tissue	- Crucial role in neural development in the adult brain, participating in the processes of differentiation, proliferation, and protection against inflammatory processes in neurons, particularly dopaminergic neurons. -Significant role in the processes of skeletal growth and maintenance of bone mass, amplifying osteoblastic activity and inhibiting osteoclast activity.	Bone loss and an increased risk of osteoporosis; PD
Parathormone (PTH) and PTH-related peptides	Parathyroid glands	- Pivotal role in regulating calcium-phosphorus and bone metabolism. PTH stimulates bone resorption by acting on the RRO pathway. - PTHrP plays a protective role in neurons against excitotoxicity and seems to be involved in modulating nerve regeneration and cerebral vasculature.	Primary hypoparathyroidism and hyperparathyroidism with related neurological and bone manifestations, such as reduction in BMD and an increased risk of osteoporotic fractures
Osteocalcin (OC)	Mature osteoblasts	- Plays a pivotal role in the mineralization process, stabilizing and facilitating the formation of mineralized bone tissue. - Is involved in the regulation of phosphate-calcium metabolism, which is essential for maintaining skeletal health. - Promotes neurite outgrowth. - Regulate a wide range of neuronal activities associated with cognitive function.	Depression; cognitive disfunction; AD
RhoA/ROCK pathway	Various tissues	- Crucial signal transduction system that plays a pivotal role in cell growth, differentiation, migration, and development. - Plays a pivotal role in neural development and survival and in regulating osteoclastic and osteoblastic activity.	Several neurological disorders; osteoporosis
Notch	Ubiquitous	Intercellular Notch signaling is crucial for diverse developmental pathways and for maintaining homeostasis in various cell types. - Plays a key role in maintaining the delicate balance between differentiation and the preservation of neural stem cells to assure the proper functioning of the CNS. - Plays a crucial role, in conjunction with the Wnt/β-catenin pathway, in regulating osteoblast differentiation.	Osteoporosis; osteosarcoma
TNF-α	Various cell types, particularly mononuclear phagocytes	- TNF-α activates cytocidal functions, playing a crucial role in the host’s defense. - TNF-α increases the expression of RANKL in bone cells, which promotes osteoclast formation and bone resorption. Additionally, it upregulates the expression of sclerostin, which contributes to bone loss by inhibiting the Wnt signaling pathway.	Neuroinflammation; PD; bone loss; Van Buchem’s disease

## Data Availability

No new data were created or analyzed in this study. Data sharing is not applicable to this article.
